# The use of human tissue surrogates in anatomical modeling for gunshot wounds simulations: an overview about “how to do” experimental terminal ballistics

**DOI:** 10.3389/fbioe.2025.1536423

**Published:** 2025-01-24

**Authors:** Lucas Meciano Pereira dos Santos, Marcelo Rodrigues da Cunha, Carlos Henrique Bertoni Reis, Daniela Vieira Buchaim, Ana Paula Bernardes da Rosa, Leandro Moreira Tempest, José Augusto Parola da Cruz, Rogério Leone Buchaim, João Paulo Mardegan Issa

**Affiliations:** ^1^ Department of Pathology and Legal Medicine, School of Medicine of Ribeirão Preto, University of São Paulo (FMRP-USP), Ribeirão Preto, Brazil; ^2^ Postgraduate Program in Health Sciences, Faculty of Medicine of Jundiaí (FMJ), Jundiaí, Brazil; ^3^ Postgraduate Program in Structural and Functional Interactions in Rehabilitation, Postgraduate Department, University of Marilia (UNIMAR), Marilia, Brazil; ^4^ Faculty of Medicine of Bauru, University of São Paulo (FMBRU-USP), Bauru, Brazil; ^5^ Medical School, University Center of Adamantina (FAI), Adamantina, Brazil; ^6^ University Center of the North of São Paulo (UNORTE), São José Do Rio Preto, Brazil; ^7^ Department of Biological Sciences, School of Dentistry of Bauru, University of São Paulo (FOB-USP), Bauru, Brazil; ^8^ Department of Basic and Oral Biology, School of Dentistry of Ribeirão Preto, University of São Paulo (FORP-USP), Ribeirão Preto, Brazil

**Keywords:** forensic pathology, ballistics, gunshot wounds, anatomic models, polymers, gelatin

## Abstract

Human tissue simulating materials are currently used in scientific research mainly because they help to avoid possible ethical issues, unlike what happens with studies involving live animals and/or human cadavers. The use of ballistic gelatin as a human soft tissue surrogate stands out, although other types of materials can be used, including polyurethane and polydimethylsiloxane in the simulation of bones and skin respectively, not to mention some computational models that completely replace the physical use of surrogate models for gunshot wound simulation. The use of human tissue surrogates can be useful in reconstructing the dynamics of a crime scene when important forensic traces cannot be found. In the absence of projectiles but in possession of the possible firearm used in the crime, for example, it is possible to verify whether the weapon in question actually fired the fatal gunshot by comparing the injury found on the victim with the injury produced on the simulant material that best represents the anatomical area impacted, as indicated in the literature. Thus, scientific advances in experimental research in terminal ballistics with tissue surrogates can positively impact applied forensic sciences in the search for better technical assistance to the justice system in solving criminal situations.

## 1 Introduction

Terminal ballistics (or wound ballistics) is the branch of ballistics science field that deals with the study of legal medical and traumatological aspects of injuries caused by firearm projectiles, also known as gunshot wounds (Humphrey and Kumaratilake, 2016; Smith and Biasotti, 1971; Holt and Kostohryz, 1983; Bolliger et al., 2016; Manta et al., 2024). A key component of laboratory terminal ballistics experiments that seek to replicate gunshot wounds is the use of properly calibrated tissue simulant materials, which mimic as closely as possible the biological properties seen in living animal tissues (Santos and Issa, 2023). This is especially true when the goal of these experiments is to confirm or evaluate how temporary and permanent cavities behave in relation to the various types of ammunition actually available and that can be used in real situations of gun violence, for example (Santos and Issa, 2023; Santos and Issa, 2024). The same importance can be observed in experimental scientific research that aims to carefully evaluate the performance of firearm projectiles and whether or not they are compatible with some quality standards established by international ammunition evaluation protocols emphasizing the real danger faced by the law enforcement agencies in which they are used (Cunha Neto et al., 2024).

In this context, the use of tissue surrogates that are not elastic (e.g., materials for sealing ducts and pipes, soap, clay, plasticine, etc.) can mislead the researcher (or even worse, such as the judicial authority in expert reports with controversial conclusions) since they exaggerate the real performance of the temporary cavity for a given ammunition and, therefore, its damaging potential (Fackler, 1988). An incorrect impression is created by this demonstration, which suggests that these cavities have the ability to destroy the victim’s tissues impacted by the projectile rather than just rupture and stretch them, which in fact could be borne by the tissues with little or no lasting damage at all (Fackler, 1988).

Based on this premise, the main objective of this review is to provide an overview of the human tissue simulating materials that are currently used for the experimental evaluation of gunshot wounds in the field of terminal ballistics studies, ranging from the simplest, such as ballistic gelatin, to the most modern and complex, such as polymers and finite element method simulation softwares, and also about the importance of these materials in aiding justice in the context of applied forensic ballistics.

## 2 Function and applicability of tissue surrogates

The use of materials that simulate human tissues (also known as tissue surrogates) is currently widely used in experimental research carried out in terminal ballistics, and among these materials are ballistic gelatin – which, by the way, is the most notable due to the large number of published scientific studies that have used it in their methodologies – and some polymers that can replace bones or skin (Mahoney et al., 2020; Bir et al., 2024; Taylor et al., 2022). Often, however, these polymers or even animal parts are associated with ballistic gelatin in an attempt to simulate the heterogeneity between tissues with a higher level of accuracy, seeking to outline the experimental configuration in a reality closer to the human organism (Bolliger et al., 2017; Mahoney et al., 2017a).

A fundamentally important factor to be considered when simulating injuries to human tissues using synthetic or artificial materials is that the tissues that make up the organism are endowed with a certain biological elasticity and, therefore, the material in question must also present, in its physical properties, characteristics that are at least similar to the type of tissue or organ that one wishes to replace in the ballistic test (Humphrey and Kumaratilake, 2016).

It is known that a firearm projectile is capable of injuring the victim’s tissues through a wounding mechanism consisting basically of the formation (and size, evidently) of temporary and permanent cavities and the fragmentation of the projectile itself or of the bones that are in its path (Cunha Neto et al., 2024; Black et al., 1941; Harvey, 1948; Hollerman et al., 1990; Barlett, 2003; Berryman, 2019; Oura et al., 2024; Watson et al., 2023). Focusing only on the cavitation mechanism as a result of a wave effect known as hydrostatic shock, the temporary cavity begins with the passage of the projectile that, when transferring its kinetic energy to the tissues, tears their fibers and moves them apart in a centrifugal direction, potentially causing damage at a distance; and then, the separated edges of the wound tend to return to their original position by elastic forces moved in a centripetal direction but, unable to collapse, result in an “empty space” or permanent cavity, which is the final form of the gunshot wound and can vary in severity depending on factors such as the tip and design of the projectile, the velocity reached in its trajectory and the anatomical region inflicted on the victim’s body (Humphrey and Kumaratilake, 2016; Holt and Kostohryz, 1983; Santos and Issa, 2024; Woodruff, 1898; Harvey and McMillen, 1947; Janzon and Seeman, 1985; Janzon et al., 1988; Watkins et al., 1988; Kieser et al., 2013; Lazovic et al., 2016; Thierauf et al., 2013; Stefanopoulos et al., 2014; Prat et al., 2017; Henwood et al., 2019; Shrestha et al., 2024).

In this case, for example, soap and clay are easily accessible and inexpensive materials that are frequently used by laypeople to picture gunshot wounds. Since these materials lack the flexibility needed to allow their results to be as close to the reality of human tissues as possible, some authors warn against using them in scientific research for the same reason (Humphrey and Kumaratilake, 2016; Santos and Issa, 2023). Soap and clay can retain the “frozen” impression of the maximum expansion brought about by the firearm projectile’s passage, making them useful tools for gaining a very brief understanding of how the wave effect works and the temporary cavity forms (Humphrey and Kumaratilake, 2016; Lewis et al., 1982). However, because they lack the elastic capacity required to permit the “wound” edges to return in a centripetal direction following the projectile’s perforation – as occurs in the human body – they are unable to provide a proper way of analyzing the permanent cavity dynamics and fail as tissue surrogates (Silvino Junior, 2020; Santos, 2022).

## 3 Ethical issues and its consequences for experimental research in terminal ballistics

A major controversy in scientific research in terminal ballistics is about what would be the ideal material to simulate the living tissues of the human body, given the existence of ethical questions regarding the use of cadavers and live animals in firearm experiments. Several studies have been published over the years with the aim of solving this situation and the overwhelming majority of them point to the difficulty that some materials have in simulating the reality of gunshot wounds in humans, especially when taking into account the heterogeneity present in the different tissues that make up the organism and its systems. To make things easier for the readers, we have prepared three information tables, two that summarizes the main differences, advantages and limitations between the types of anatomical modeling as discussed in the present opportunity (Tables 1, 2), and another to serve as a guide for interested researchers or forensic pathologists depending on the type of artificial material they have at hand (Table 3). Nonetheless, in order to avoid any trampled comprehension, we strongly advise reading the text before concentrating on the tables.

**TABLE 1:** Summary of the major variations regarding type and application of human tissue surrogate materials in anatomical modeling for terminal ballistics scientific research.

Material type	Overall usage considerations
Live animals or parts of dead animals	Pigs are the gold standard animal for terminal ballistics studies that do not use artificial materials in their methodologies, since, surprisingly, they are the animals that most closely resemble humans anatomically. Due to ethical issues about the use of live animals, it is recommended to use parts obtained from animals slaughtered for legal sale in markets (the fresher, the better)
Organic-based ballistic gelatin (bare)	Ballistic gelatin (especially in its 10% concentration) is considered the gold standard material for experimental studies in the scientific field of terminal ballistics. Proposed in the middle 1980s by Fackler and Malinowski, the methodological standardization of this material is very important to eliminate statistical biases from the final results of ballistic tests that may use this type of anatomical modeling
Animal mixed ballistic gelatin blocks	Studies with live animals are practically impossible nowadays due to ethical impediments in justifying their sacrifice in experiments with firearms. To get around this issue, some researchers associate animal parts obtained from butchers or meatpackers – preferably of porcine origin – with ballistic gelatin blocks in an attempt to add some tissue heterogeneity to anatomical models used in experimental simulations for ballistic tests
Fabric-coated ballistic gelatin	The use of certain types of clothing as barriers between the firearm and the ballistic gelatin block can influence the formation of the temporary cavity. In cases like this, the temporary cavity may occur “early” and a greater amount of kinetic energy is received by the target, especially if the projectile reaches high velocity levels (≥609.6 m/s or 2.000 ft/s)
Synthetic polymers	Polymeric materials are used in scientific research in terminal ballistics to increase the complexity in the heterogeneity of the tissues that will be reproduced. Some associations between synthetic polymers are very useful for studies that seeks to evaluate gunshot wounds in the head and neck region through anatomical head models, such as polyurethane (bone surrogate) and polydimethylsiloxane (skin surrogate), for example. However, research in this area does not completely abandon the use of ballistic gelatin, which is normally used at a 10% standard to simulate brain tissue
Finite element analysis	With the finite element method (FEM), approximate mathematical solutions to real-world problems (such as gunshot wounds) can be found through the use of numerical differential equations. In the context of terminal ballistics, the researcher can perform a gunshot without even touching a firearm or reloading ammunition. The main disadvantage is the learning curve and the high investment required for its implementation
Human cadavers	Some authors argue that studies using ballistic gelatin or pigs and other animals are biased, always highlighting the same problem, that is, the lack of fidelity in representing a whole human body. Consequently, they recommend studying what they consider to be the closest thing to a living human being: a human cadaver (preferably in fresh conditions). The main problem is that, for some countries, such as Brazil, for example, in addition to the serious ethical issues that shooting a corpse only for experimental purposes would cause, the difficulty of obtaining the resources needed to implant this type of methodology in national territory (e.g., efficient donor programs, cadaver transportation in good conditions for freezing and thawing, etc.) turns it almost utopian nowadays

**TABLE 2:** Brief summary of the advantages and limitations of human tissue simulating materials.

Material	Key properties	Elasticity	Reproducibility	Ethical considerations
Human cadavers and pork legs (fresh)	Both are considered very close to the anatomical configuration of living human tissues under ideal conditions	Damaged by cold *post mortem* storage conditions and the putrefaction time elapsed	Virtually impossible, since there cannot be two corpses or parts of dead animals that are exactly the same in terms of rigorous standardization	Problematic, since the justification for the use of cadavers and animals in scientific research involving gunshots may be very difficult or even impossible in some situations
Ballistic gelatin	Material very similar to human soft tissues. Alternating between concentrations of 10%–20% allows the simulation of different organs	If properly standardized, it allows good assessments of the temporary and permanent cavities dynamics	Easily achieved through the use of standardized formulas and ideal conditions for preparation and material storage	Minimum, since its use eliminates the need for humans and animals or the handle of materials that poses chemical or biological risks
Polyurethane and polydimethylsiloxane	Best choice for simulating human bone or skin respectively	Patterns for tissue disruption and bone fragmentation are similar to real gunshot wounds characteristics, which may aid in forensic situations of headshot executions, for example	It depends a lot on the manufacturer and the market price, which can be a major limiting factor	It can be very difficult if conflicts such as the destination of waste from its manufacturing process or its disposal are taken into consideration, since these are plastic materials after all
Finite element method	Eliminates the use of real firearms or ammunition through the use of computers and simulation software	Data on the elasticity or deformation of the tissues that will be “impacted” in the simulation must be entered as mathematical information for it to be effective	It depends a lot on the operator’s learning curve and whether the software used by operators at different research institutions is the same or have similar calibration	Minimal, since it does not involve the use of animals, humans or biohazardous materials. However, there may be some conflicts as to whether medical record data should be accessed to include information in the software

**TABLE 3:** Guidelines for the use of organic-based or synthetic simulants in terminal ballistics.

	Calibrated	Non-calibrated
Organic-based (10% or 20% ballistic gelatin)	1. It can be used in scientific research. However, it is recommended to always check the calibration of the block before taking samples, even if used for the preparation of animal parts embedded in ballistic gelatin	2. Its use in scientific research is no longer recommended, but it can still be an excellent material for sports practice in shooting clubs
Synthetic	3. It can be used in scientific research, especially in the simulation of more complex anatomical models. It is necessary to verify whether the cost of producing or obtaining the synthetic material will exceed the research budget or hinder the pace of sample collections and limit the final statistics	4. Don’t even bother using synthetic materials whose calibration cannot be verified or traced back to the manufacturer’s information in scientific research. The high cost of production may also contraindicate their use for recreational target shooting activities

Moreover, if necessary, any ethical approval for terminal ballistics research should address the following framework: 1) brief introduction to the guidelines and recommendations on research ethics in the legislation in force in the country where the study will be carried out; 2) what is a Research Ethics Committee and what is its importance; 3) the complete data of the researcher and/or research institution and, if applicable, the participant (as in the case of surveys or reports involving victims recently injured by gunshot wounds); 4) the justification of the project (some bibliographical references may be cited to strengthen the consent form, but as long as overly technical terms that could confuse the research participant or the evaluator in the Ethics Committee are avoided); 5) excerpts that make clear what the objectives, risks, disadvantages, benefits and advantages of the research are; 6) there can be no doubt that the conduct of the research will not result in any payment in cash, gifts or other forms of reward to anyone involved with its ethical validation; 7) that there will be compensation guarantees if data that allows the identification of those involved in the research is leaked, even without the intent of causing damage; and, most importantly, 8) make it clear that the results obtained will be used for academic and scientific dissemination purposes and may be present in class materials, lectures and other scientific events. Further adjustments will need to be made in accordance with the requirements of Research Ethics Committees in different countries.

## 4 Experimental studies with animal parts

Many studies in the past used whole animals to represent the human body in the investigation of gunshot wounds, but the large animals that were most commonly used (such as goats and pigs) made the research expensive and required large physical plants for their care and feeding, which made the progress of the work extremely difficult (Lewis et al., 1982; Callender, 1943). Research that utilizes animal tissues (especially pigs) in its materials and methods has the advantage of allowing for the observation of events that are extremely close to reality, making it possible to consider the variety that exists in human anatomy (however imperfectly) (Komenda et al., 2013; Jennings et al., 2018; Rickman and Shackel, 2019). Analysis of the rate at which skeletal muscles deform in response to the damage brought on by the temporary cavity can be done at this level of inquiry (Stefanopoulos et al., 2019).

We can see from a variety of studies why pigs are used as standard experimental animals when conducting research whose methodology does not involve artificial materials to replicate gunshot wounds (Janzon and Seeman, 1985; Janzon et al., 1988; Henwood et al., 2019; Nicholas and Welsch, 2004; Rodrigues et al., 2018; Varlet et al., 2020). Due to its great anatomical similarity with humans (especially regarding soft tissues), pigs were used extensively in ballistics research in this context up until the end of the 1980s, which also marked the development of standardized recipes used in the manufacturing of ballistic gelatin blocks (especially its 10% concentration), thereby reducing the possibility of statistical biases resulting from the use of materials that are naturally non-standardized (Humphrey and Kumaratilake, 2016; Janzon and Seeman, 1985; Janzon et al., 1988; Nicholas and Welsch, 2004; Rodrigues et al., 2018; Fackler and Malinowski, 1985). Thus, the pig gradually disappeared from the methodologies of studies in terminal ballistics that were published in subsequent years and, currently, it is practically impossible (or at least unlikely) for a scientific research project that involves the use of live pigs in this area, as in the past, to be approved by any Research Ethics Committee without a very well-elaborated justification (Santos, 2022), despite some rare exceptions can be observed (Yoganandan et al., 2024a; Yoganandan et al., 2024b; Yoganandan et al., 2024c).

Researchers have resorted to using animal parts obtained from a butcher or meatpacker or from legalized hunting activities, depending on the legislation in force in the country where the research is being conducted, since the use of live pigs in scientific research has become too difficult and methodologically impracticable (Santos and Issa, 2023). If the researcher wishes to use animal parts in his terminal ballistics studies, the use of pork leg (hind portion or “shank”) is highly recommended for human comparison (Santos and Issa, 2023). The part must be removed from a pig that preferably weighs between 50 and 70 kg (±110 and 154 lb) to obtain good results that can be extrapolated to real situations of gunshot wounds in humans, even though many studies differ on this subject (Janzon and Seeman, 1985; Janzon et al., 1988; Nicholas and Welsch, 2004; Fackler et al., 1984a; Fackler et al., 1984b). In this area, the literature lacks research that seeks to effectively evaluate the existence of statistically significant differences between the use of hind legs obtained from male and female pigs in matters involving terminal ballistics (Santos and Issa, 2023). On the other hand, although it is a topic that needs to be further investigated in future research, there also appears to be a preference for using hind legs or other parts from female pigs weighing between 60 and 80 kg (±132 and 176 lb), since these animals may have a distribution of fatty tissue that allows better comparisons of gunshot wounds in human soft tissue (Santos and Issa, 2023; Jennings et al., 2018).

Nevertheless, a major problem with using pigs or any other type of animal of this size (or even larger) is the methodological standardization that scientific studies require. In other words, a study is statistically valid when its materials and methods are very well standardized. Thus, for a study involving pigs or pork parts, the animals should be strictly standardized if the samples were planned to be collected in a repeat-style methodology for standard deviation statistics, for example. Following this line of reasoning – and exaggerating a little, if we may – in a study in terminal ballistics that proposes to evaluate shots in swine hind legs, several shots with the same type of ammunition and caliber should be carried out in the same anatomical point (which is already virtually impossible) in ideally identical hind legs obtained from pigs that have fed on the same food throughout their lives, having the same age, the same weight, raised in the same environmental conditions and so on.

Therefore, even with the preference for research on pigs, it is practically unfeasible to standardize a serious study using only animal parts because all results will be influenced by the experimental setup and the standardization of these animals, which means that the final statistics would be extremely difficult to apply to real scenarios of gunshot wounds in humans (and that is what really matters in research with tissue surrogates in terminal ballistics), regardless of the number of samples collected.

## 5 Ballistic gelatin (bare blocks)

The hydrolytic breakdown of collagen protein produces the natural polymer known as “gelatin” (Yarahmadi et al., 2024). After collagen isolation, gelatin can be obtained in tablet, granule or powder form through acid or alkaline hydrolysis, and must usually be dissolved in water before use (Yarahmadi et al., 2024). The powder form can be obtained by pretreating bovine or swine carcasses in acidic or alkaline solutions, resulting in type A or type B gelatin, respectively (Cunha Neto et al., 2024; Pullen et al., 2022). The resulting strength and stiffness are designated by the “bloom number,” with the best consistency recommended for the production of ballistic gelatin being type A powder with 250 bloom (Humphrey and Kumaratilake, 2016; Santos and Issa, 2024; Pullen et al., 2022; Fackler and Malinowski, 1988). There are several levels of bloom number of ordnance gelatin currently available, basically ranging from 50 to 300 (Humphrey and Kumaratilake, 2016). For that reason, researchers should pay close attention to the manufacturer’s information. Some gelatin powders found in supermarkets, better known as “colorless flavorless gelatin,” widely used in food and drug industries, have a low bloom number, usually between 140–180, and are contraindicated for the production of ballistic gelatin because they result in uncalibrated blocks whose “softness” will invariably cause an exaggeration in the damage caused by the firearm projectile.

The bloom number will directly affect the levels of strength and stiffness of ballistic gelatin blocks, but it will not be solely responsible for these two characteristics in the final product, as both also depend on the concentration of the powder in relation to the amount of water used in the formula and the temperature of the mixture during preparation (Humphrey and Kumaratilake, 2016). First of all, it is recommended to mix the gelatin powder in cold water, pouring the powder on top of the water and never the other way around, stirring well and letting the final mixture rest for 2 h (preferably inside a refrigerator or other cold controlled environment) in order to completely hydrate de gelatin particles and homogenize the liquid/powder medium (Fackler and Malinowski, 1985; Fackler and Malinowski, 1988). After the resting time, the gelled mixture must be heated over low heat or in hot water bath (i.e., “bain-marie”) to prevent the material from burning. By changing the proportion between powder and water, concentrations of 10% and 20% can be obtained. In one way or another, certain critical conditions will affect the block’s competence to pass the scientific calibration test, such as temperature and curing time (Pullen et al., 2022; Maiden et al., 2015).

The literature reports that 1921 was the year that introduced the first scientific work with ballistic gelatin, where the researcher used blocks at 20% standard and found a way to implant small cotton threads inside them that were displaced with the passage of the projectiles, thus enabling the visualization of the damaging pattern (Lewis et al., 1982; Wilson, 1921). Over the years, several publications have scientifically validated the use of ballistic gelatin (Humphrey and Kumaratilake, 2016; Bir et al., 2024). However, it is said that ballistic gelatin has the unfortunate disadvantage of being a uniform material and this is why its results must be interpreted with the utmost care due to the heterogeneity of the human body and its dimensions, which is made up of muscles, bones, tendons, fat, among other tissues arranged in a continuous solution, despite being an excellent material for simulating soft tissues (Humphrey and Kumaratilake, 2016; Santos and Issa, 2023; Santos and Issa, 2024; Cunha Neto et al., 2024; Bir et al., 2024; Santos, 2022; Carr et al., 2018). In more specific words, ballistic gelatin is ideal for representing soft tissues only, as a sort of “general replacement” for these tissues together, but fails to represent harder tissues such as bones, tooth enamel, cartilage and joints, for example.

**FIGURE 1:**
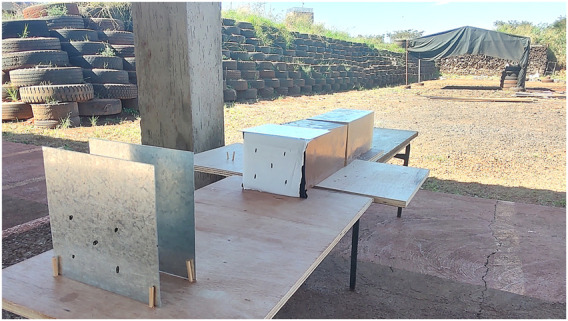
Two blocks of 10% ballistic gelatin placed to evaluate gunshot shielding situations.

Ballistic gelatin has been criticized for a number of reasons, but it is important to note that a tissue simulator’s properties do not have to match those of real tissue in the human body as long as the outcomes can be measured, appropriately extrapolated and scaled to the reality it is meant to replicate (Santos and Issa, 2023; Jussila, 2004). The researcher should always wait at least 36 h (preferably 48–72 h) after putting the liquid mixture into a mold, which will be kept at 4°C in a temperature-controlled area, before using the ballistic gelatin block (Santos and Issa, 2024; Cunha Neto et al., 2024; Nicholas and Welsch, 2004; Fackler and Malinowski, 1988; Fackler et al., 1988). After that, the ballistic test can be conducted with the desired ammunition, weaponry or experimental configuration (Figure 1).

**FIGURE 2:**
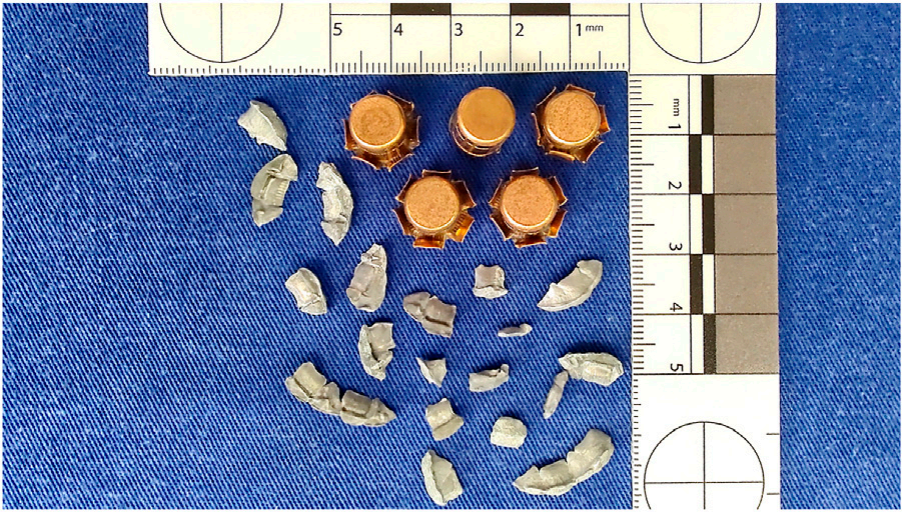
Five .357 Magnum projectiles recovered from 10% ballistic gelatin.

In general terms, there are four types of ballistic “phenomena” that can be observed in ballistic gelatin: 1) the smooth reduction in the velocity of the firearm projectile upon striking the gelatin block; 2) full penetration potential, where the projectile may yaw and change its path (yawing bullet effect); 3) the rolling or overturning of the projectile due to the resistance (drag) provided by the internal environment of the gelatin block and a yaw greater than 90° (tumbling bullet effect); and 4) the expansion or stretching (i.e., temporary cavity) and compression or crushing (i.e., permanent cavity) of the impacted tissues, culminating in the final stabilization of the wound path taken by the projectile in the very form of a gunshot wound (Humphrey and Kumaratilake, 2016; Santos and Issa, 2023; Jennings et al., 2018; Rodrigues et al., 2018; Zecheru et al., 2018; Riva et al., 2018; Wen et al., 2017). Examining the deformation and fragmentation patterns of the projectile when it lodges inside the ballistic gelatin block – a situation in which it lacks the force required to pass through it – is another possible option (Figure 2).

To put it in another way, when used as a human soft tissue model, ballistic gelatin is the best material for gradually illustrating the wounding effects of a firearm projectile (Breeze et al., 2014). By capturing high velocity images, for example, the researcher can see the projectile in the very moment when it hits the ballistic gelatin block, the maximum size of the temporary cavity, or the final form of the permanent cavity, along with the lodged projectile deformation and/or fragmentation or passed through along the block’s length at depth (Breeze et al., 2014).

It is important to note that the primary purpose of ballistic gelatin is to visualize the profile of the temporary and permanent cavities of the simulated gunshot wound, allowing an adequate approximation to human soft tissues (Nicholas and Welsch, 2004; Carr et al., 2018). The two main ballistic gelatin standards currently in use are the 20% at 10°C, also referred to as NATO gelatin, and the 10% at 4°C, suggested by Fackler and Malinowski in 1985 (Fackler and Malinowski, 1985) and validated in the sequence of scientific meetings that followed the infamous 1986 FBI Miami shootout events (Humphrey and Kumaratilake, 2016; Cunha Neto et al., 2024; Guey et al., 2018; Schyma, 2020; Wang et al., 2015). Since there is no recognized NATO standard for gelatin, it is recommended to refer to it as 20% gelatin (by mass), even though the “NATO gelatin” name has become widespread for the 20% ballistic (or ordnance) gelatin concentration (Carr et al., 2018). Despite some authors make comparisons between the two standards, it has been reported that 20% ballistic gelatin is stiffer than 10% ballistic gelatin and that results of projectiles depth of penetration are shorter in 20% concentration (Carr et al., 2018; Mabbot et al., 2016). For any 10% or 20% ballistic gelatin block, both the permanent and temporary cavity will have scientific validation with real human soft tissue comparison only if properly standardized. Each concentration type of ballistic gelatin has its own calibration methods, normally evaluating the penetration range of 0.177” (4.5 mm) steel BBs inside the blocks whose formula is intended to be calibrated (Bir et al., 2024; Pullen et al., 2022; Guey et al., 2018). To validate 10% ballistic gelatin blocks, five shots must be fired at a distance of 10 feet (approx. 3 m) between the muzzle of the firearm and the block to be calibrated, at a velocity range of 590 ± 15 ft/s (approx. 180 ± 5 m/s), with the steel BBs penetrating and remaining lodged at an average interval of 2–15/16″ to 3–3/4″ (or 85 ± 10 mm) (Santos and Issa, 2024; Cunha Neto et al., 2024). As for 20% ballistic gelatin blocks, the recommendation is to fire 0.177″ copper-plated spherical BBs at a velocity range of 590 ± 15 ft/s, but at a distance of 6.5 feet (approx. 2 m), and the copper-plated BBs should have a penetration depth of 1–7/16″ to 1–29/32″ (or 42.5 ± 6 mm) (Bir et al., 2024). As for block size, Fackler and Malinowski’s original recommendation for 10% standard (which can also be used for 20%) is 25 × 25 × 50 cm (Fackler and Malinowski, 1985), although the use of 15 × 15 × 40 cm blocks obtained by using Clear Ballistics’ FBI Block Mold^®^ to ensure the latter measurements have showed good results (Santos and Issa, 2024; Cunha Neto et al., 2024). Polymeric materials such as PermaGel™ and Clear Ballistics^®^ are examples of synthetic “ballistic gel” blocks that might not be appropriate for use in scientific research since literature has reported different results compared to organic-based ballistic gelatin, including some evidence of ageing after remelting processes and burned portions inside the blocks (Cunha Neto et al., 2024; Carr et al., 2018). Nonetheless, a very recently published study concluded that Clear Ballistics^®^ synthetic gelatin blocks at a concentration of 20% is an “acceptable” tissue simulant as long as standard calibration methods are followed (LeSuer et al., 2024).

**FIGURE 3:**
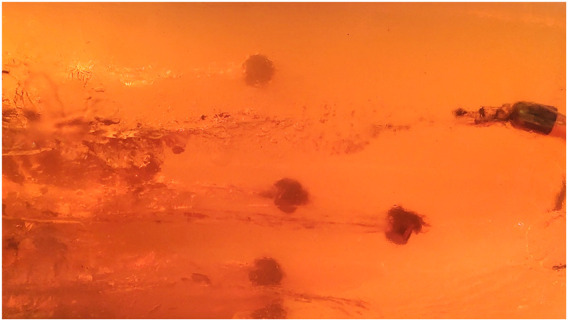
Five .40 S&W projectiles lodged inside a 20% ballistic gelatin block.

**FIGURE 4:**
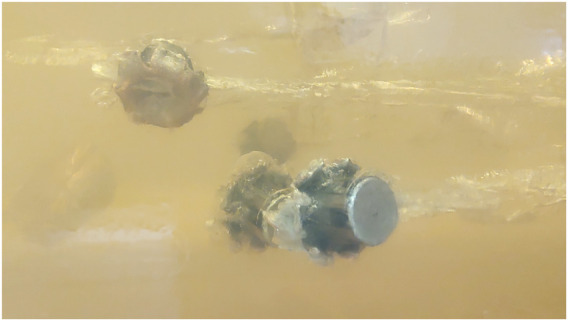
Four .40 S&W projectiles lodged inside a 10% ballistic gelatin block.

As the element that enables the observation of events that a firearm projectile may be subjected to (e.g., deformative expansion, fragmentation, yawing, tumbling), as well as the various patterns of formation of the temporary cavity and its final configuration in a permanent cavity, the translucency of a ballistic gelatin block, whether in a concentration of 20% (Figure 3) or 10% (Figure 4), is crucial for the use of this type of material in scientific studies (or even in expert reconstructions of forensic pathology cases) (Santos and Issa, 2023).

Two groups of researchers will always be most active in ballistic gelatin research: one aims to increase the quality and lethality of the ammunition under test (commonly industries with an interest in military research), while the other aims to comprehend the detrimental effects of the projectiles on the human body in order to increase its capacity for gunshot survival (Carr et al., 2018). Research aiming to investigate the many forms of gunshot wound care, surgical methods for eliminating projectiles or their fragments, and advancements in protective material technologies for the creation of military apparel and vehicle armor are all excellent examples in this final category (Santos and Issa, 2024). Additionally, ballistic gelatin is a material that can also be used for shooting practice in purely recreational activities.

## 6 Ballistic gelatin mixed with animal parts

During the gelatin block-making process, dead animal parts (the fresher, the better) are combined with ballistic gelatin, normally at a 10% standard concentration. This kind of material was developed due to the difficulty of bare ballistic gelatin in accurately replicate the variability between the human body’s tissues (Lewis et al., 1982). A well-known example in literature is when pig lungs or ribs are submerged in a ballistic gelatin block to mimic bullet wounds to the thorax (Bolliger et al., 2017; Rodrigues et al., 2018). Similarly, recent research methodologies using this type of anatomical modeling have been documented in the last years literature, including the embedding of adult deer femurs inside ballistic gelatin blocks, as well as pig legs, scapulas and hemothoraces, or even the use of pig leather cutouts for the external coating of the blocks, among many other examples (Kieser et al., 2013; Thierauf et al., 2013; Zecheru et al., 2018; Pircher et al., 2019; Kerkhoff et al., 2018; Mahoney et al., 2017b; Zhang et al., 2015). The use of synthetic ballistic gel to replace soft tissue in mixed models with bone specimens for fracture experiments has also been reported recently (Schwab et al., 2024; Biehl et al., 2024).

Most studies involving the use of ballistic gelatin blocks mixed with animal organs or tissues seek to evaluate the harmful effects of temporary cavitation in bones when caused by high-velocity projectiles (≥609.6 m/s or 2.000 ft/s) such as 5.56 × 45 mm and 7.62 × 49 mm or 7.62 × 51 mm calibers and to what extent the so-called “distant fractures” are possible in different situations of angle, distance, type of projectile, etc., where the shot is fired aiming at the block of gelatin, but in such a way that the projectile does not hit the animal part embedded in the block directly but passes close by, causing only the temporary cavity to touch it when it expands in a centrifugal direction.

In these terms, a study with an animal part mixed gelatin model has revealed that bone fractures can, in fact, result from the temporary cavity’s maximum expansion affecting the most fragile part of the bones (and maybe the same is possible when previously healed fractures were present), which is particularly true when the projectile passes very close to the embedded bone (2 cm or less), creating a wedge-shaped fracture with a high risk of contamination – a factor of crucial importance for surgeons contemplating the need of surgical intervention in hospitalized victims with gunshot wounds (Zhang et al., 2015).

**FIGURE 5:**
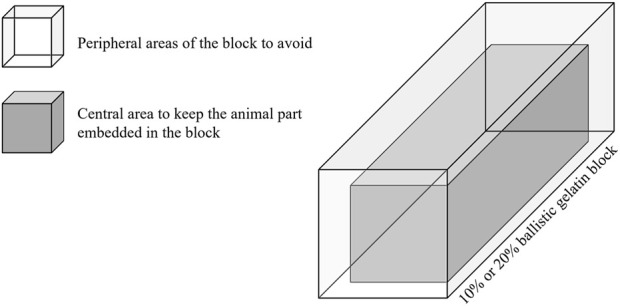
Graphic representation of the best way to position animal parts in ballistic gelatin.

When collecting samples with animal part mixed gelatin, it is important that the animal part (especially if it is a bone) remains fixed in the central portion of the block. Avoiding the peripheral regions of the ballistic gelatin block, even if the bone or organ is translocated three-dimensionally in terms of height, width or depth, keeping the animal part “centralized” allows the stress caused by the temporary cavity effects to be distributed to it in a more homogeneous dynamic. To prevent any piece of the animal part from remaining without suffering the effects of temporary cavitation, it is recommended that all portions be embedded so that no surface is exposed outside the block when it is ready for use (Figure 5).

The piece can also be placed in the most posterior part of the block (in depth), opposite the side that will receive the shot, to simulate situations in which the projectile has to pass through a certain amount of soft tissue to reach some organ or noble structure, such as in ballistic wounds from thoracic and abdominal trauma (Rodrigues et al., 2018).

Achieving the animal part mixed ballistic gelatin model is relatively simple, but some difficulties are precisely related to what the researcher must do to keep the animal piece centered in the block. Obviously, the samples of animal’s tissues or organs must be placed in the blocks before gelatinization took place, but the researcher must wait until the exteriors of the molds are just a little bit warm to the touch before putting the samples in to avoid excessive heating of the tissues if the gelatin formula that was chosen calls for pouring hot water (Lewis et al., 1982). One way to make the animal organs get stuck in the middle of the ballistic gelatin block is as follows: 1) the organs may be attached, for example, to thin, flat lead weights by using 3–0 silk suture to hold them in place (since most tissues float in the gelatin medium before it solidifies); 2) the samples are held in place while the gelatin hardens by these weights, which sink to the bottom of the mold; 3) after the block have fully gelatinized, the flat weights are removed from the inverted block surface and the sutures extracted using a forceps (or maybe a needle holder) (Lewis et al., 1982).

Outside the field of experimental terminal ballistics, this type of anatomical modeling has proven useful in replacing soft tissue for cone-beam computed tomography imaging, where researchers embedded a pig’s head in ballistic gelatin and verified this possibility (Nascimento et al., 2021). Additionally, an interesting case report showed an episode in which a different kind of animal part mixed gelatin model was used in a real forensic situation. In the case in question, a hunter was shot in the back by accident, and the two shooters who were with him were suspected of inadvertently shooting the victim. The specialists used tissue simulators to conduct some ballistic tests in an attempt to distinguish between the two suspect choices because the lethal projectile had passed through the body and was not discovered at the crime scene. The victim’s chest was simulated by two 15 mm layers of 10% ballistic gelatin interposed by a pig lung wrapped in a plastic bag, and shots were performed with the suspects’ weapons. Following an analysis of the simulated injuries in the hybrid model, the method identified one of the hunters, whose position and rifle/ammunition combination matched the victim’s gunshot wounds and the probable firing angle (Bresson and Franck, 2010).

## 7 Ballistic gelatin coated on clothing fabrics

The way to acquire this model is as simple as possible. It can be easily obtained by simply making a block of standard ballistic gelatin and covering it with the fabric that must be evaluated in the experimental setup, as shown in Figure 1, where the face of the block that will receive the gunshot was covered with a layer of light fabric (white) disposed on top of a layer of heavy fabric (black). This type of anatomical modeling is performed to simulate a situation where the gunshot wound victim is wearing clothes, as is most often the case.

By associating ballistic gelatin with any type of fabric or clothing as a coating, a more complex reality is simulated, even for low-velocity projectiles (<609.6 m/s or 2.000 ft/s). The main finding with this model is the change in the moment of formation of the temporary cavity, that is, the “anticipation” of its point of maximum expansion in relation to the impacted target compared to the situation in which there was no coating at all (Santos and Issa, 2023).

It makes sense to assume that wearing a certain type of clothing will increase protection against gunshot penetration. However, studies in this area have shown that the materials of clothing, particularly military apparel, can influence the moment at which the temporary cavity expands to its greatest extent, causing more damage to the victims than if they were naked or wearing lighter clothing (Stevenson et al., 2019). Clearly, the type of projectile (or another blunt/perforating instrument) and armor protection has a significant impact on this kind of result as well (Santos and Issa, 2023; Chaufer et al., 2024; Kislov et al., 2022).

In other words, a firearm projectile that could easily pass through a limb might yaw or even tumble much earlier when it comes into contact with clothing acting as a barrier before it reaches the victim’s body, increasing the temporary cavity’s destructive intensity because of its anticipation, which can even cause the limb’s amputation (Prat et al., 2017; Mahoney et al., 2017b; Stevenson et al., 2019; Weisenbach et al., 2018; Wightman et al., 2015). Even if ballistic armor stops the bullet, it can still transfer residual kinetic energy to the tissues underneath, causing what is known as behind armor blunt trauma (or BABT), and this type of damage can cause severe, incapacitating, or even deadly injuries (Prat et al., 2017; Yoganandan et al., 2023; Bustamante and Cronin, 2024; Bass et al., 2006; Lange et al., 2024).

This does not mean that the soldier must carry out the mission with the rifle in hand but without clothes for his greater safety. In fact, the vast majority of research that used blocks of ballistic gelatin associated with clothing and that observed this “early formation” phenomenon of the temporary cavity, evaluated projectiles fired in high-velocity, mainly in the 7.62 × 51 mm caliber (Santos and Issa, 2023). Nevertheless, it should be clear in mind that a huge part of gunshot wounds observed in everyday life are those caused by low-velocity projectiles (Jakoi et al., 2015; Pinto et al., 2019). Thus, the soldier is most of the time protected when wearing the standard uniform with the clothing or ballistic armor and helmet that is normally provided to him (Santos and Issa, 2023). Prevention is always the best course of action, even though the risk outweighs the benefits in some situations. Moreover, terminal ballistics research is continuously being conducted around the world, and the outcomes can be applied to industrial projects aimed at creating more effective protective materials.

## 8 Synthetic polymeric materials

Polymer-derived materials are inserted into the context of terminal ballistics through much more complex simulations that seek to evaluate injuries where the reproduction of tissue continuity solutions (e.g., between muscle and bone) is essential to obtain results worthy of extrapolation to real situations (Santos and Issa, 2023). Polymeric materials that are capable of acting as tissue surrogate in simulations of the human skull or other soft tissue regions with underlying bone support are good examples. The association of models with military clothing and protective materials such as ballistic armor and helmets is also very common in this scientific field. Because it can reproduce human bone tissue with a high degree of similarity, polyurethane is, in general, the most used polymer in this type of anatomical modeling (Pircher et al., 2019; Mahoney et al., 2019a; Mahoney et al., 2019b; Mahoney et al., 2018; Taylor and Kranioti, 2018).

Some polymers are often used together, such as polydimethylsiloxane for simulating skin and polyurethane for simulating mineralized bone tissue (Mahoney et al., 2017b; Mahoney et al., 2019a; Mahoney et al., 2018). When combined, these two polymers enable a notable expansion of the information obtained from experimental sample collections. Fragmentation analysis – of the projectile or bone – and the aftermath of residues in the polyurethane, as if it were the cortical bone layer in situations of shots to the head with the gun muzzle pressed against the skin, for example, can even be applied in real forensic circumstances, as long as the conditions observed in crime scene analysis and in the victim’s examination by the forensic pathologist are respected and compared, of course (e.g., the victim’s position in relation to the firearm, ammunition used, angle of the shot, etc.) (Taylor and Kranioti, 2018). However, despite the macroscopic similarity of polyurethane as a bone surrogate, there are microscopic dissimilarities with real bone tissue that must be taken into account (Smith et al., 2015).

In summary, synthetic polymeric materials have the advantage of simulating some situations in which ballistic gelatin is limited by its homogeneity, such as bones and soft tissues of the human body. However, the standardization of their complex manufacturing process and the difficulty in obtaining the technology necessary for the production of these polymers, which are factors that consequently result in their high market value, are also limiting factors for their application in scientific research (Santos and Issa, 2023).

Although synthetic polymers can closely simulate the human head and neck or thoracic regions, what we see in the literature is that some studies in terminal ballistics that involve the use of polymeric materials end up using ballistic gelatin in one way or another, which is normally used to simulate brain tissue in head models by introducing the material in liquid phase (especially in the 10% standard) through the foramen magnum of polyurethane skulls (Mahoney et al., 2019a; Mahoney et al., 2018).

The fact that new studies propose the use of artificial synthetic materials, but on the other hand do not completely disregard the application of organic-based ballistic gelatin, reinforces the thesis that, even with all its criticisms and certain disadvantages, ballistic gelatin is an essential material (and even indispensable in some situations) for experimental simulations of gunshot wounds.

## 9 Computer simulations with finite element analysis

The finite element method (FEM) is a mathematical analysis that consists of the discretization of a continuous medium – while maintaining its original properties – into small elements described by differential equations and solved by mathematical models (Lotti et al., 2006). The origin of the development of this resource, that is, the principle that the physical behavior of structures could be described mathematically through the use of differential mathematical equations, occurred in the 18th century with scientists such as Gottfried Wilhelm Leibniz (1646–1716) and Johann Bernoulli (1667–1748), but its application viability was only possible with the advent of modern computers (Lotti et al., 2006; Cadfem, 2024).

It is known that anatomical models simulated by FEM can be very valuable tools for the study of blunt injuries in the thoracic and abdominal regions, repeatedly identified as the most common regions of the human body for cases of injuries caused by firearm projectiles (Lazovic et al., 2016; Jakoi et al., 2015; Pinto et al., 2019; Shen et al., 2008; Holmes, 1952; Peonim et al., 2016). Using the concepts learned from forensic traumatology, the injury caused by a firearm projectile is an injury with perforating and blunt characteristics and, therefore, belongs to the class of injuries caused by mechanical energy through the combination of kinetic energy with potential energy. Thus, a gunshot wound only occurs because the projectile is capable of pass through the victim’s tissues (perforating component), since it hits them with enough energy to do so (blunt component), and depends largely on the inherent characteristics of the projectile, the weapon that fired it and the anatomical region of the human body that will be impacted (Santos and Issa, 2024). That being the case, if anatomical modeling with FEM is a good evaluator of blunt trauma, it is also advisable to apply it in the study of gunshot wounds (Bustamante and Cronin, 2024).

Through a flexible and useful analysis, studies in terminal ballistics through FEM computational approximations allow adjustments and different types of adaptation for anatomic models created in software. A variety of shooting distances and angles using another variety of projectiles in a third variety of impacted tissues can be tested, as long as precise information is available about each one, such as, for example, the characteristics of the material used to make the firearm projectile that will be evaluated (in addition to other information in the manufacturer’s possession, some of which are extremely difficult or impossible to access due to patent issues), average microhardness of the bone that will be impacted in the simulation, and so on (Santos and Issa, 2023).

Particularly in the simulation of soft tissue/internal organs and anthropometric values, it is important to take into account the limitations of the model when interpreting results obtained from FEM modeling (Humphrey and Kumaratilake, 2016; Chu et al., 1994; Cobetto et al., 2018). Since every experimental configuration is generally simulated under the same conditions, results should be assessed in a comparative rather than an absolute manner (Cobetto et al., 2018). The main advantage of using FEM models is the considerable reduction of ethical issues compared to the use of human cadavers (Chaufer et al., 2023).

Although it is not a simple methodology because it requires a high investment value, confidential information from ammunition manufacturers and a high degree of calibration, both of the software and the researcher (i.e., the “learning curve”), studies using FEM computational approximations greatly reduce the issues from Research Ethics Committees – mainly in the field of terminal ballistics – since they do not only dispense the use of animals and cadavers, but also the need for shooters and the consequent signing of informed consent forms by them (Santos and Issa, 2023). In FEM models, for example, it is possible to test several hypotheses and experimental configurations, such as shooting angle and distance, projectile velocity or other performances in external ballistics and dynamism of the impacted tissues (Santos and Issa, 2023). In this type of situation, the researcher can obtain a real-time gunshot without even using a real gun (Zhang et al., 2015; Bracq et al., 2019; Costa et al., 2017; Jin et al., 2019).

## 10 Research on human cadavers

In a scientific context, since the animal-human extrapolation in terminal ballistics, as seen previously, is debated in terms of sample bias, studies with human cadavers should then be elected as the gold standard for research that aims to obtain results that are applicable for comparative purposes with humans. However, this is not what happens. If there is no well-established and efficient body donor program for handling fresh cadavers, the application of the research methodology becomes very difficult, mainly due to the decay of tissues as putrefaction progresses, not to mention other problems such as the severe ethical impediment that may exist for the use of cadavers in experimental research (as in Brazil, for example).

One must always consider the important ethical question of sacrificing an animal purely and exclusively for research whose results may be considered irrelevant or biased (Varlet et al., 2020). Furthermore, just as animal-human extrapolation is questionable, the extrapolation of specific organs and tissues to the entire human body is also questionable, this being one of the main factors that motivated studies on cadavers over the years (Varlet et al., 2020).

Despite all the ethical issues and its use having already been discouraged in the literature (Humphrey and Kumaratilake, 2016), human cadavers are also an option to replace living human tissues in terminal ballistics research. But some risk factors must be highlighted, such as transportation, for example, that, if necessary, will normally involve freezing and thawing processes, which can negatively affect the final results due to changes in the biomechanics of the tissues that would be subjected to testing (Koser et al., 2022).

When researching human injuries and ways to mitigate them, the mechanical characteristics of soft tissues are crucial (Singh and Chanda, 2021). It is known that poor soft tissue behavior and bone fragility are the main disadvantages when dealing with dead bodies (Nouma et al., 2016). Nevertheless, the use of anesthetized pigs and human cadavers was compared in the investigation of BABT trauma, where it was observed that shots fired at cadavers always presented a greater degree of severity (Prat et al., 2012). On the other hand, for example, differences were observed in cranial backspatter patterns when shots were performed on the head of human cadavers infused with bovine blood and blood-soaked sponges, where shots on cadavers were able to reproduce bloodstains of different sizes due to the heterogeneity of human tissues compared to the sponge, a homogeneous simulant commonly used in controlled backspatter experiments (Rossi et al., 2018).

Since obtaining repeated data related to experimental gunshot wounds in cadavers is hampered by the tissue alteration caused by putrefaction, one solution observed was the analysis of the effect of putrefaction itself on projectiles placed in different parts of dead bodies obtained from donation programs and how this could affect the examination of the projectile in forensic ballistics after its removal days later (Bolton et al., 2024). Post mortem human subjects (PMHS) of human skulls were also used to investigate the injury mechanism of blast-induced trauma to the brain, where their cranial contents were discarded and replaced by ballistic gel (Salzar et al., 2017).

In short, for the acquisition of repeatable data from experimental gunshots against a given target, ballistic gelatin appears to outperform the use of cadavers (Santos and Issa, 2023). While human or animal cadavers are subject to a variety of factors that act on both the decomposition and preservation of their tissues (Varlet et al., 2020; Koser et al., 2022), which could influence research results with statistical biases depending on the methodology used, 10% organic-based ballistic gelatin is a fully standardizable and reproducible material (Cunha Neto et al., 2024), not to mention the possibility of its reuse in a formulation that is actually under patent consideration in Brazil (Santos, 2022; Santos and Issa, 2021).

## 11 Conclusion

The secret of a good surrogate material is its proximity to human tissues, and the more real the “replacement”, the better for experimental tests of gunshot wound simulation. Some materials have the advantage of returning results to the researcher whose data are easily applicable in stronger statistical analyses due to repeatable data, such as ballistic gelatin, for example, especially in its 10% standard, which can also be associated with other types of anatomical modeling. Moreover, there seems to be a tendency for future research in terminal ballistics to be stimulated by computational FEM models and artificial intelligence, although they are composed of complex methodologies that may demand from the researcher a hard skill with new information technologies and a high level of financial investment.

Recent studies still support the idea that scientific experiments in terminal ballistics with human tissue surrogate materials are fundamental and indispensable in many ways, especially due to its high potential for contribution with several sectors of society, from improvements in surgical techniques and trauma prevention to the development of new military technologies.

Perhaps some readers of this work – and we know that the scientific community in terminal ballistics is quite rigorous – may think that the emphasis given to ballistic gelatin was quite repetitive, but it was done that way because it is the current gold standard for human soft tissue surrogate material. It is very important to further investigate the relationships between ballistic gelatin concentrations and other types of materials for simulating living human tissue, whether synthetic or organic-based. These investigations are nothing new in the literature, but perhaps they can point the way to a completely different new gold standard that could help us to better understand the secrets of preventing and treating gunshot wounds.

Among the types of tissue surrogates discussed, ballistic gelatin is the easiest and safest to handle, especially among researchers in the early stages of postgraduate studies or supervisors who are still setting up an experimental ballistics laboratory and still have few or absolutely no resources at all. The very possibility of associating it with animal parts or coating it with clothing fabrics and even varying the concentration between the standards of 10% (“FBI/Fackler”) and 20% (“NATO”), as long as the standards for scientific standardization are respected, are already more than enough to bring a wide range of methodological variation for the purpose of providing scientific researches with good levels of complexity (as suggested by most academic institutions of higher education worldwide), which can still be increased if the research is associated with the experimental reproduction of real cases in applied forensic ballistics or by changing, in the same methodology, the elements of weaponry, ammunition and shooting distance, leading to an even greater range of options for materials and methods. However, if resources were abundant, a foray into more expensive materials is highly recommended, since there is plenty of room in the current ballistics literature for scientific research with synthetic polymers and computational approximations with FEM. Nevertheless, if this is the case, the researcher must be aware of the possibility of having greater problems with issues such as standardization and reproducibility. Finally, if the country’s Ethics Committees and its current legislation on research ethics allow it, there are even more gaps to fill for ballistics research with human cadavers, which is by far the least explored scenario currently.
